# Initial insight into the function of the lysosomal 66.3 kDa protein from mouse by means of X-ray crystallography

**DOI:** 10.1186/1472-6807-9-56

**Published:** 2009-08-25

**Authors:** Kristina Lakomek, Achim Dickmanns, Matthias Kettwig, Henning Urlaub, Ralf Ficner, Torben Lübke

**Affiliations:** 1 Department of Molecular Structural Biology, Institute of Microbiology and Genetics, GZMB, Georg-August University Goettingen, Justus-von-Liebig-Weg 11, D-37077 Goettingen, Germany; 2 Center of Biochemistry and Molecular Cell Biology, Department Biochemistry II, Georg-August University Goettingen, Heinrich-Dueker-Weg 12, D-37073 Goettingen, Germany; 3 Bioanalytical Mass Spectrometry Group, Max Planck Institute for Biophysical Chemistry, Am Fassberg 11, D-37077 Goettingen, Germany

## Abstract

**Background:**

The lysosomal 66.3 kDa protein from mouse is a soluble, mannose 6-phosphate containing protein of so far unknown function. It is synthesized as a glycosylated 75 kDa precursor that undergoes limited proteolysis leading to a 28 kDa N- and a 40 kDa C-terminal fragment.

**Results:**

In order to gain insight into the function and the post-translational maturation process of the glycosylated 66.3 kDa protein, three crystal structures were determined that represent different maturation states. These structures demonstrate that the 28 kDa and 40 kDa fragment which have been derived by a proteolytic cleavage remain associated. Mass spectrometric analysis confirmed the subsequent trimming of the C-terminus of the 28 kDa fragment making a large pocket accessible, at the bottom of which the putative active site is located. The crystal structures reveal a significant similarity of the 66.3 kDa protein to several bacterial hydrolases. The core αββα sandwich fold and a cysteine residue at the N-terminus of the 40 kDa fragment (C249) classify the 66.3 kDa protein as a member of the structurally defined N-terminal nucleophile (Ntn) hydrolase superfamily.

**Conclusion:**

Due to the close resemblance of the 66.3 kDa protein to members of the Ntn hydrolase superfamily a hydrolytic activity on substrates containing a non-peptide amide bond seems reasonable. The structural homology which comprises both the overall fold and essential active site residues also implies an autocatalytic maturation process of the lysosomal 66.3 kDa protein. Upon the proteolytic cleavage between S248 and C249, a deep pocket becomes solvent accessible, which harbors the putative active site of the 66.3 kDa protein.

## Background

In order to spatially separate the vast number of divergent reactions carried out by intracellular enzymes, eukaryotic cells are compartmentalized into several membrane-bound organelles. Among these organelles, the lysosomal compartment contains more than 50 hydrolases required for degradation of macromolecules or even whole organelles entering the lysosome by endocytotic or autophagic pathways [1,2] (reviewed in [3]).

This degradation process and thus the hydrolases involved are essential for the cell as reflected by the manifestation of severe diseases which are characterized by the accumulation of undigested substrates in the lysosome due to the lack of hydrolytic enzyme activities. The associated pathogenic phenotypes are collectively referred to as "lysosomal storage disorders" (reviewed in [4]). However, the lysosomal compartment does not only serve as a digestive compartment but also plays a key role in many other cellular processes like modulation of peptide hormones and bioactive lipids, tissue homeostasis, inflammation [5-8] as well as neuroprotection [9]. Furthermore, lysosomes are involved in the pathogenesis of Alzheimer disease [10], autoimmune diseases and in the initiation and progression of cancer [11].

Recently, several proteome studies of the lysosomal compartment have identified a considerable set of novel lysosomal proteins. Most of these sub-proteomic studies took advantage of a specific carbohydrate modification of newly synthesized soluble lysosomal proteins, the mannose 6-phosphate residue (M6P) [1,2,12-16] as reviewed in [3]. *In vivo*, M6P-containing proteins are recognized by mannose 6-phosphate receptors (MPRs) at the trans-Golgi network (TGN) and transported to endosomes, in which the receptor-ligand complex dissociates due to the acidic pH. Finally, the M6P-containing proteins are delivered to lysosomes, while the MPRs return to the TGN. In most lysosomal sub-proteome analyses, M6P-containing proteins were purified by affinity chromatography on immobilized MPRs and subsequently analysed by mass spectrometry based techniques.

One novel protein that was identified in tissues derived from mouse, rat and human was referred to as hypothetical 66.3 kDa protein [14-16].

Subsequently, the murine 66.3 kDa protein [17] and its human ortholog p76 [18] were characterized in more detail regarding their lysosomal localization, processing and glycosylation status. The maturation of the orthologs from mouse and human includes both, limited proteolysis and the usage of all five or six potential N-glycosylation sites. The murine 66.3 kDa protein is synthesized as glycosylated preproprotein of about 75 kDa in apparent molecular mass. After the co-translational removal of the N-terminal signal peptide, the remaining proprotein is sorted to the lysosomal compartment and matures into a 28 kDa N-terminal fragment and a 40 kDa C-terminal fragment [17]. A similar processing was described for the human ortholog p76 resulting in a 32 kDa N-terminal fragment and a 45 kDa C-terminal fragment [18]. The same authors suggested an additional maturation step for the 40 kDa fragment from mouse into a C-terminal 27 kDa fragment. Such a limited proteolysis in the endosomal/lysosomal compartment is a common hallmark of lysosomal hydrolases and a prerequisite to their hydrolytic activation [19]. These proteins are commonly synthesized as preproenzymes. The signal sequence is removed during their synthesis into the lumen of the endoplasmic reticulum resulting in the corresponding proenzymes. These precursors are most often processed by limited proteolysis in late endosomes or lysosomes and thus converted into their hydrolytically active forms. By this kind of processing, an activity of the enzyme at the site of translation – within the endoplasmic reticulum -, which might harm cellular components, is prevented.

The 66.3 kDa protein is conserved among vertebrates and shows homology to the Lamina ancestor precursor of *Drosophila melanogaster *[20] (29% identity for 416 aligned residues), the ribonuclease P protein subunit p30 of *Entamoeba histolytica *[21] (30% for 349 aligned residues from C249 to I505), phospholipase B from *Dictyostelium discoideum *[22] (39% identity for 518 aligned residues) as well as to the highly glycosylated integral membrane protein p67 from *Trypanosoma brucei *(33% identity for 473 aligned residues) [17,18,23]. The trypanosomal protein p67 has recently been demonstrated to be essential for maintenance of normal lysosomal structure and physiology in bloodstream-stage cells [24]. In contrast, no homologous proteins have been found in yeast and prokaryotes.

Neither bioinformatics analysis nor the detailed characterization of the mouse lysosomal 66.3 kDa protein and its human ortholog p76 have provided any hint regarding the activity and the physiological function. Recently, we determined the three-dimensional structure of the mouse 66.3 kDa protein [25]. Here we report three structures of the 66.3 kDa protein that represent different maturation states of the post-translational processing. By limited proteolysis, a 28 kDa and a 40 kDa fragment are derived. They stay associated forming a compact entity, and the C-terminus of the 28 kDa fragment is further trimmed. The obtained results were substantiated by mass spectrometric analysis. Furthermore, the 66.3 kDa protein could be assigned to the superfamily of N-terminal nucleophile (Ntn) hydrolases. Despite the lack of a significant sequence similarity there is a close resemblance to several bacterial hydrolases regarding the protein fold and residues forming the catalytic centre. Additionally, a detailed comparison of the three crystal structures of the 66.3 kDa protein reported in this work with the homologous structures provides initial insight into its catalytic activity and suggests a mechanism of the enzyme's activation by autocatalytic proteolysis.

## Methods

### Data collection and structure determination

The glycosylated 66.3 kDa protein from mouse was produced by overexpression in the human fibrosarcoma cell line HT1080 and purified as described [17] except for some minor modifications that are summarized below. Since the 66.3 kDa protein and its proteolytic 28 kDa and 40 kDa fragments could not be separated by affinity and ion exchange chromatography and gel filtration, the mixture containing all three polypeptide chains was used for crystallization. The protein was crystallized under acidic conditions, and the structure was solved at 2.40 Å by means of sulphur SAD phasing using long wavelength radiation [25]. The three data sets described in the following had been collected prior to the sulphur SAD experiment. At a shorter wavelength (0.8141 Å), a data set was collected from the same crystal that was used for the sulphur SAD (data set "xe1h", PDB-ID 3FGR) on the BESSY beamline BL-14.2, which was equipped with an SX165 detector (Rayonics LLC, Illinois, USA), and processed with HKL2000 (HKL Research, Inc., Charlottesville, VA). Two additional data sets had been collected previously from a native crystal (data set "native", PDB-ID 3FGT) and from a crystal soaked with potassium iodide (data set "KI", PDB-ID 3FGW). The original purification protocol, which was used for the protein batches leading to the native and KI soaked crystals, was lacking a gel filtration. This final step was only applied for the protein preparation used for the SAD phasing and the determination of the structure 3FGR.

The crystal, on which the native data set was collected, was grown under the previously described conditions [25], whereas the crystal for the KI data set was obtained under slightly different conditions. Instead of Tris/HCl pH 8.0 [25], the concentrated protein was dissolved in a buffer system of sodium chloride and sodium phosphate buffer pH 7.4. Furthermore, the crystallization drop was composed of 0.7 μl of protein solution (23 mg/ml) and reservoir each (12% (w/v) PEG 4000, 100 mM NaAc/HAc pH 4.6, 100 mM NH_4_Ac). Thus, the final salt concentration was slightly reduced by about 19% and Tris was exchanged by a phosphate buffer system.

The data sets "native" and "KI" were collected on the DESY beamline X13 (DESY, Hamburg, Germany), which was equipped with a marccd165 detector (Marresearch GmbH, Norderstedt, Germany), and on the BESSY beamline BL-14.1 (BESSY, Berlin, Germany) on a marmosaic225 detector (Marresearch GmbH, Norderstedt, Germany), respectively. The images were integrated with XDS [26] and Mosflm [27], respectively, and scaled using SCALA of the CCP4 program suite [28]. The iodide soaked crystal severely suffered from radiation damage. However, a 97% complete data set with a reasonable R factor of R_p.i.m. _= 9.2% could be obtained. The three structures derived from the different data sets were solved by means of Molecular Replacement with MOLREP [29] using the 2.4 Å structure of the 66.3 kDa protein (3FBX) as a search model [25]. The 1.8 Å and 2.4 Å structures were manually completed by cycling between REFMAC5 of the CCP4 program suite and COOT [30], while CNS [31,32] and COOT were used for the 2.8 Å structure. Data collection and refinement statistics for the three structures are summarized in Table 1.

**Table 1:** Summary of crystallographic data. R_p.i.m. _= precision-indicating R factor, R_merge _= merging R factor

**PDB-ID**	**3FGR (xe1h; cleaved)**	**3FGW (KI; uncleaved)**	**3FGT (native; cleaved)**
data set	xe1h	KI	native

wavelength (Å)	0.91841	1.80000	0.80150

number of images	305	300	406

oscillation steps (°)	0.5	0.5	0.4

space group	C 1 2 1	C 1 2 1	C 1 2 1

cell [Å, °]	148.74	146.69	145.57
	89.56	88.11	88.22
	64.81	73.55	63.27
	β 98.68	β 111.10	β 98.10

resolution range^a ^(Å)	50.00–1.70 (1.76–1.70)	46.07–2.80 (2.95–2.80)	30.00–2.40 (2.53–2.40)

completeness (%)	99.5 (96.2)	97.2 (96.2)	99.8 (100.0)

redundancy	3.2 (2.6)	3.0 (3.0)	3.4 (3.4)

unique reflections (rejections)	91,683 (164)	21,117 (418)	31,031 (3,487)

R_sym_* or R_p.i.m._^# ^(%)	3.3 (41.9)*	9.2 (28.9)#	6.1 (29.4)#

I/sigma	32.1 (2.4)	5.6 (2.0)	9.5 (3.5)

X-ray source	BL-14.2	BL-14.1	X13

**Refinement statistics**			

amino acids in asu (chain)	524:	529:	524:
	V63-T238 (A)	P61-N239 (A)	P60-N239 (A)
	G245-S248 (A)		
	C249-P592 (B)	G245-D594 (A)	C249-P592 (B)

molecules in asu	1	1	1

resolution (Å)	29.26–1.80	46.07–2.80	29.49–2.40

R_work_^e^	15.2	22.3	16.6

R_free_^f^	18.2	24.9	20.7

number of non-H atoms			
protein	4396	4211	4275
water	576	176	299
solvent	78	107	90

rmsd^g^			
bonds (Å)	0.015	0.005	0.012
angles (°)	1.533	0.983	1.493

average B factors	24.3	37.0	28.1

### Structure analysis

Four structures of the 66.3 kDa protein were refined and were deposited within the Protein Databank. The structure 3FBX has been solved by SAD and is published elsewhere [25]. This work describes structures of the cleaved forms 3FGR (xe1h) and 3FGT (native) as well as of the "uncleaved form" 3FGW (KI).

The final 1.8 Å structure of crystal form I (PDB-ID 3FGR) includes 524 amino acid residues. While V63-T238 and G245-S248 belong to the polypeptide chain A, C249-P592 form the continuous chain B. Additionally, five N-glycans are included in the final structure. Two N-acetylglucosamine (NAG) moieties are linked to N115 and N441 each, while only one NAG moiety each could be placed at N93, N236 and N520. One xenon atom that had been caught in a hydrophobic pocket during a soak in a xenon gas chamber [25] and one sodium ion as well as two acetate anions from the crystallization buffer and eleven glycerol molecules from the cryo protecting solution are included in the solvent model. SIOCS (version 2007/07 alpha_test 0.1; Heisen & Sheldrick, in preparation) was used for prediction of the amide/imidazole orientations of asparagine, glutamine and histidine side chains. The final structure was refined to R factors of R_work _= 15.2% and R_free _= 18.2% with a FOM of 0.90. The stereochemical analysis of the refined structure with PROCHECK [33] detected two proline residues (P502 and P592) as well as one aspartate residue (D316) to exhibit a *cis *peptide conformation and six residues with torsion angles outside the expected Ramachandran regions (M275, S306, N394, R401, Y431 and H577).

In contrast to the structure 3FGR, the native 2.4 Å structure (PDB-ID 3FGT) comprises three additional residues at the N-terminus (D60-P62) and one extra residue in the intermediate region of the sequence, namely N239, but lacks four amino acids at the C-terminus of chain A (G245-S248). Chain B contains the same residues as in 3FGR resulting in altogether 524 amino acids in 3FGT (D60-N239, C249-P592). Four NAG moieties are attached to the residues N115 (2), N236 (1) and N441 (1), respectively. Three acetate anions as well as five glycerol, one triethylene glycol and two tetraethylene glycol molecules are included in the solvent model.

The structure derived from the KI derivative crystal (PDB-ID 3FGW) includes the residues P61-N239 and G245-D594. In contrast to 3FGR, G245-S248 are connected to C249. The structure 3FGW contains five NAG moieties and one mannose (MAN) moiety (1 NAG each at N93, N236 and N441 as well as 2 NAGs and 1 MAN at N115). Furthermore, the solvent model comprises three glycerol molecules, seven iodide anions and one sodium ion.

The N-terminal amino acids L47 – P59/D60/P62 (3FGT/3FGW/3FGR), N239/T240 (3FGR/3FGT+3FGW) – L244/S248 (3FGR+3FGW/3FGT) as well as the C-terminal residues (W593, D594 (3FGR, 3FGT)) and the eleven residues of the C-terminal affinity tag (GRGSHHHHHHG)) are missing due to the lack of unambiguously interpretable electron density. However, the residues N239-S246 and N239-S248, respectively, which are located in a functionally important region, have been shown to be belonging to the 28 kDa fragment by means of mass spectrometry as outlined below.

Superpositions for the determination of root mean square deviations (r.m.s.d.s) between two structures as well as for graphical comparison were performed with the program SUPERPOSE of the CCP4 program suite using the superposition of specified atoms if possible (for 3FGR, 3FGT and 3FGW) and secondary structure matching for less related structures (e.g. lysosomal AGA). For superposition with the about 330 amino acids containing enzymes PVA and CBAH, only chain B of the 66.3 kDa protein was used (344 aa), while the whole molecule served as the reference for the larger structures of cephalosporin acylase (CA) and penicillin G acylase (PGA) (557 residues). The differences between the three structures that concern four loops of the 28 kDa fragment connecting the β-strands β1 and β2, β2 and β3, β4 and α1, and α-helices α1 and α2, respectively, are based on distinct intermolecular crystal contacts with symmetry equivalent protein molecules, in which the loops are involved. Calculations of the electrostatic surface potential were performed with DELPHI 4.1 [34].

### In-gel digestion of the 66.3 kDa protein and the processed fragments and mass spectrometry (MS)

The purified 66.3 kDa protein was incubated under crystallization conditions (3FGT) and treated with N-Glycosidase F (PGF) (Roche, Mannheim, Germany) according to the protocol. 10 μg non-treated and PNGase treated samples were separated by 1D PAGE (NuPAGE, Invitrogen, Karlsruhe) and proteins were Coomassie stained (G250). Visible bands were cut out and proteins were in-gel digested with endoproteinase Trypsin according to [35]. Peptides were extracted and analyzed by liquid chromatography (LC) coupled tandem mass spectrometry (MS/MS) on an Orbitrap XL (Thermo Fisher Scientific, Schwerte, Germany) under standard conditions, i.e. collision induced dissociation (CID) in the linear ion trap (LIT). MS and MS/MS product ion spectra were searched against NCBInr database containing the full-length FASTA sequence of the 66.3 kDa protein and both processed fragments, i.e. the N-terminal 28 kDa and the C-terminal 40 kDa fragment, using MASCOT as search engine. MS and MS/MS spectra were further manually evaluated for tryptic peptides derived from PNGase treated sample harbouring the C-terminus of the N-terminal 28 kDa fragment and eventually shortened versions (238–248 TDTKPSLGSGS, 238–247 TDTKPSLGSG, 238–246 TDTKPSLGS, 238–245 TDTKPSLG).

### Figure preparation and preparation of Additional files

**Figure 1:**
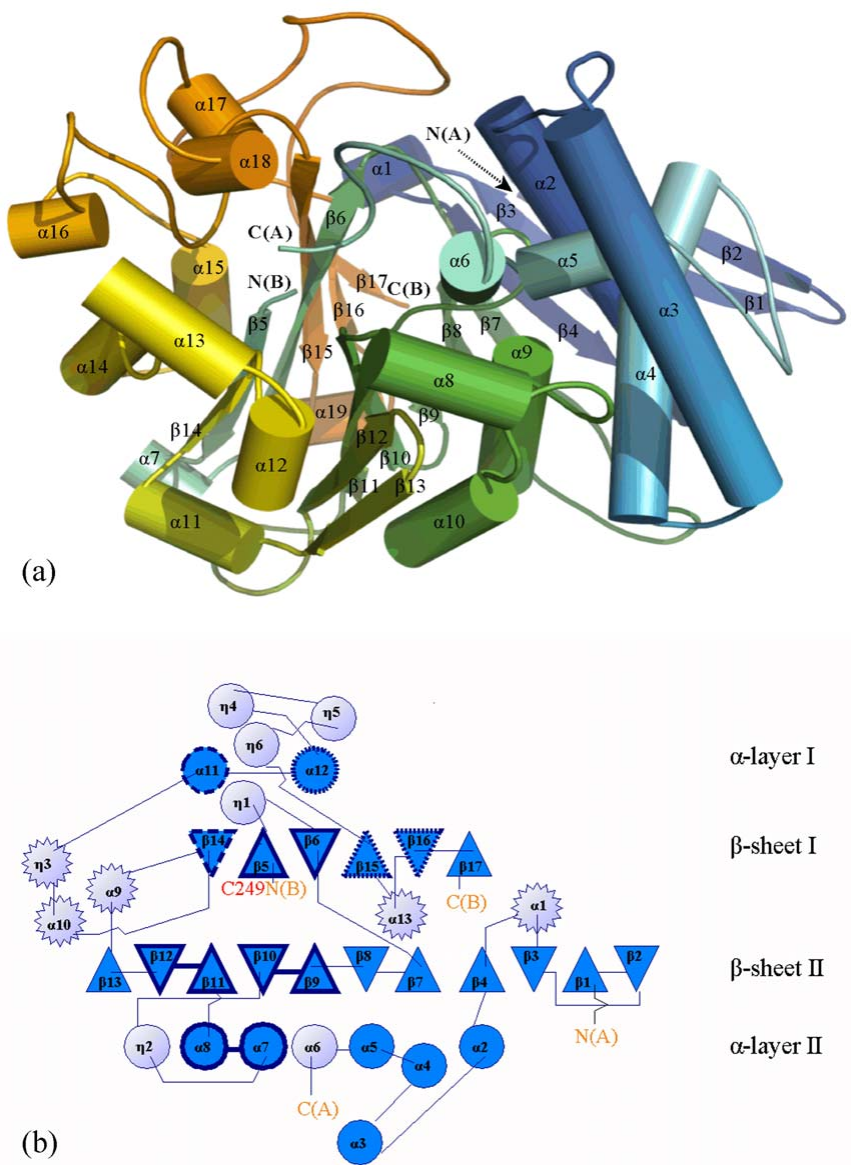
**Overall structure of the 66.3 kDa protein from mouse**. (a) The residues are rainbow-coloured according to their position in the polypeptide chain from the N-terminus (blue) to the C-terminus (orange) and represented in cartoon mode with smoothed loops. (b) In the topology diagram, light blue circles represent helices below or above the anti-parallel β-sheets, on either side of them, blue circles show helices which sandwich the sheets, and light blue stars display helical structures in loops above or below the sandwich between both β-sheets. N(A), C(A), N(B) and C(B) mark the N- and C-termini of the 28 kDa and 40 kDa fragment, respectively.

**Figure 2:**
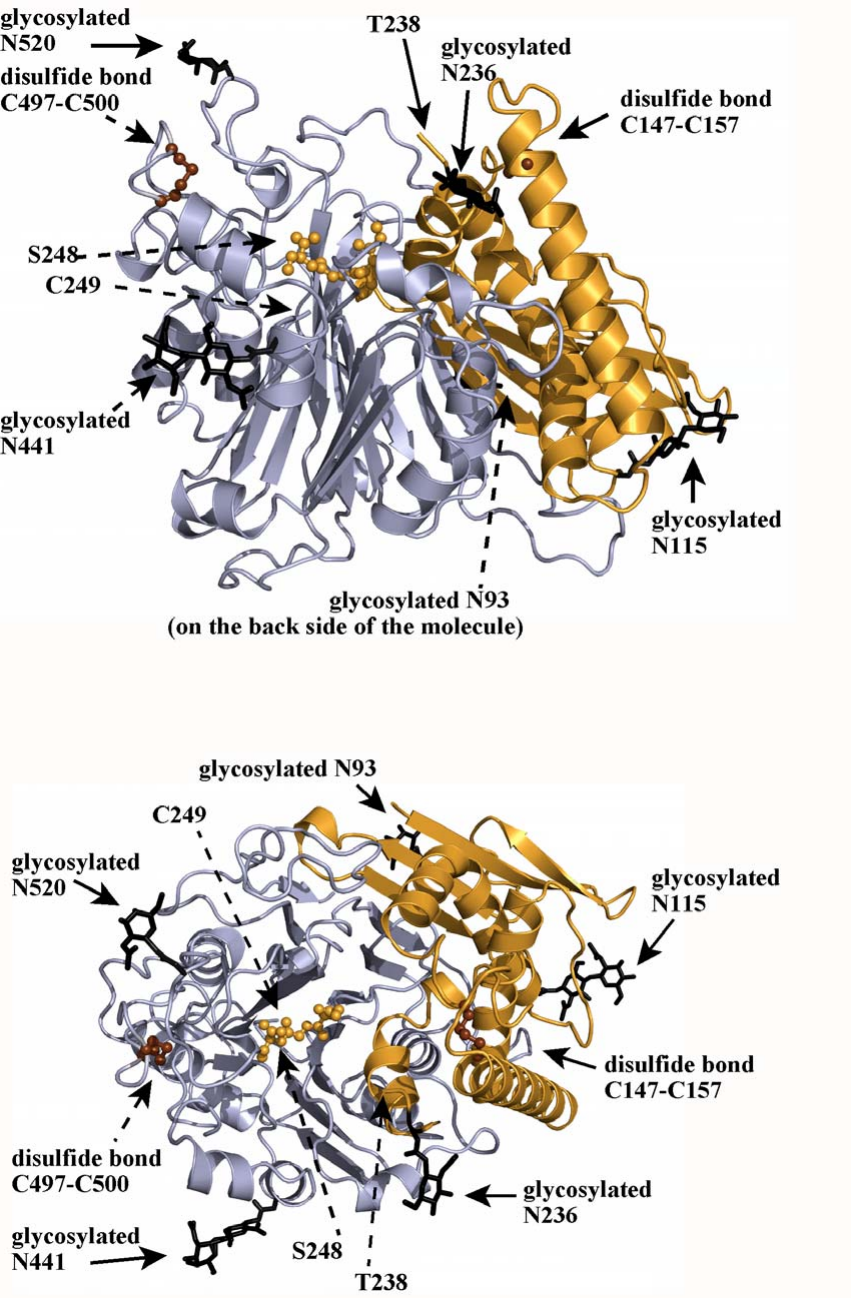
**Cartoon model of the 66.3 kDa protein (3FGR) viewed along the β-sheets (at the top) and from the top (after a turn by 90°) (at the bottom)**. The 28 kDa and 40 kDa fragment are coloured in orange and blue, respectively. The last four C-terminal residues of the 28 kDa fragment (G245-S248) as well as the two intramolecular disulfide bonds are highlighted in ball and stick mode and coloured in orange and brown, respectively. The five glycans and the asparagine residues, at which they are attached, are shown as thick black lines.

**Figure 3:**
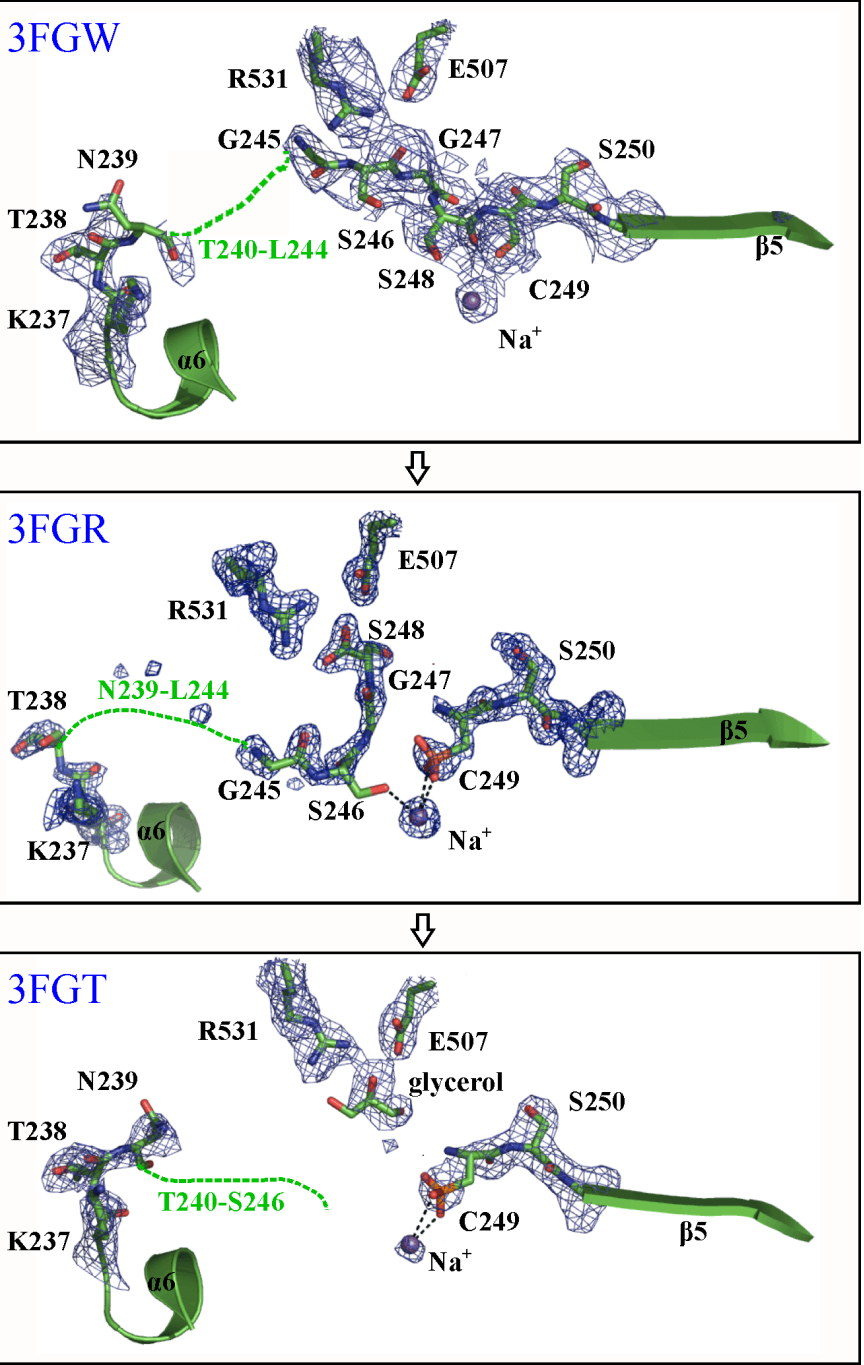
**Comparison of the region of the 66.3 kDa protein, which differs significantly between the one chain and two chain variants**. The structures are shown in the putative order of the maturation process starting at the top. The most significant differences concern the residue range N239-C249. This range and additionally the adjacent residues K237, T238 and S250 as well as the side chains of E507 and R531 and a glycerol molecule are shown in stick mode with the surrounding electron density of a F_o_F_c _simulated annealing omit map at a contour level of 5.0 s (carbon, oxygen and nitrogen atoms in green, red and blue, respectively). The bound Na^+ ^ion is shown as a blue sphere. Interactions between the sodium ion and C249 or S246 are indicated by black dashed lines. For orientation, L231-N236 and A251-K254 are shown in cartoon mode.

**Figure 4:**
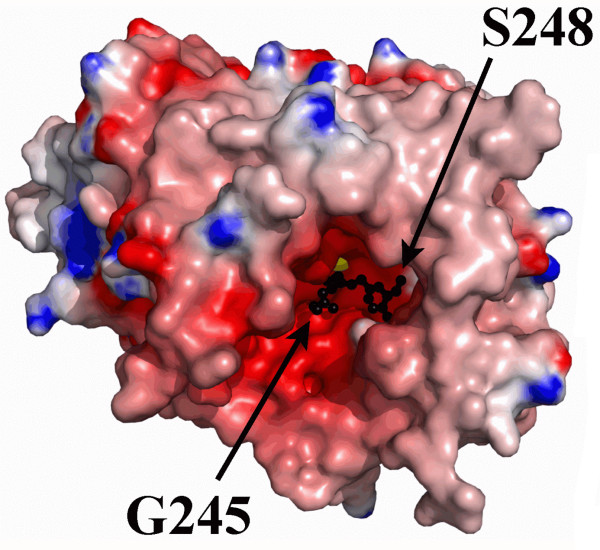
**Electrostatic surface potential of the 66.3 kDa protein (3FGR)**. The residue ranges V63-T238 and C249-P592 are shown as surfaces and coloured according to their electrostatic potential with positive and negative charges in blue and red, respectively. The residues G245-S248 are shown in ball and stick mode and coloured in black. The bound Na^+ ^ion is represented as a yellow sphere.

**Figure 5:**
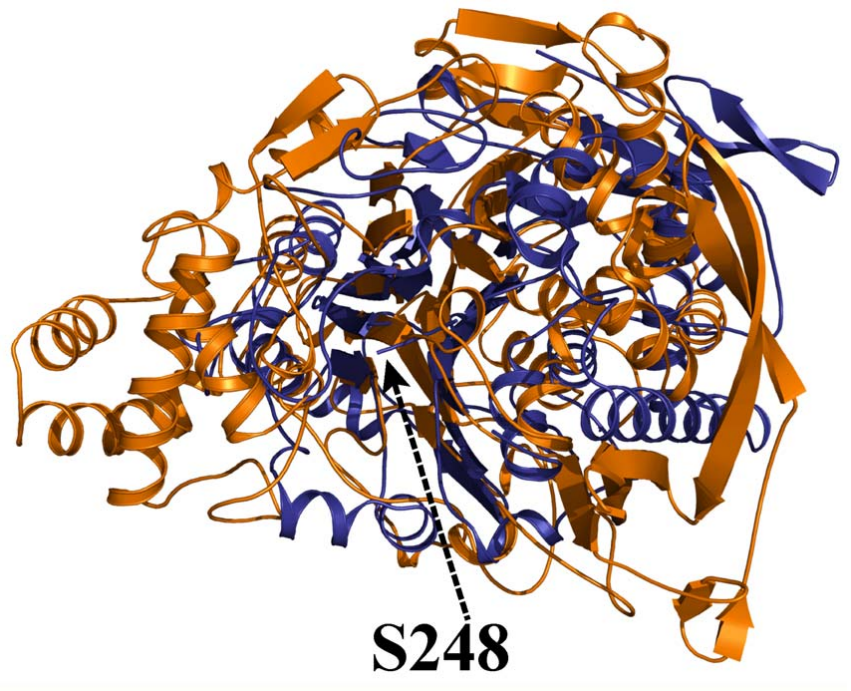
**Superposition of the 66.3 kDa protein with cephalosporin acylase**. The structures of the 66.3 kDa protein (3FGR) and cephalosporin acylase (1OQZ) are shown in cartoon mode and coloured in blue and orange, respectively.

**Figure 6:**
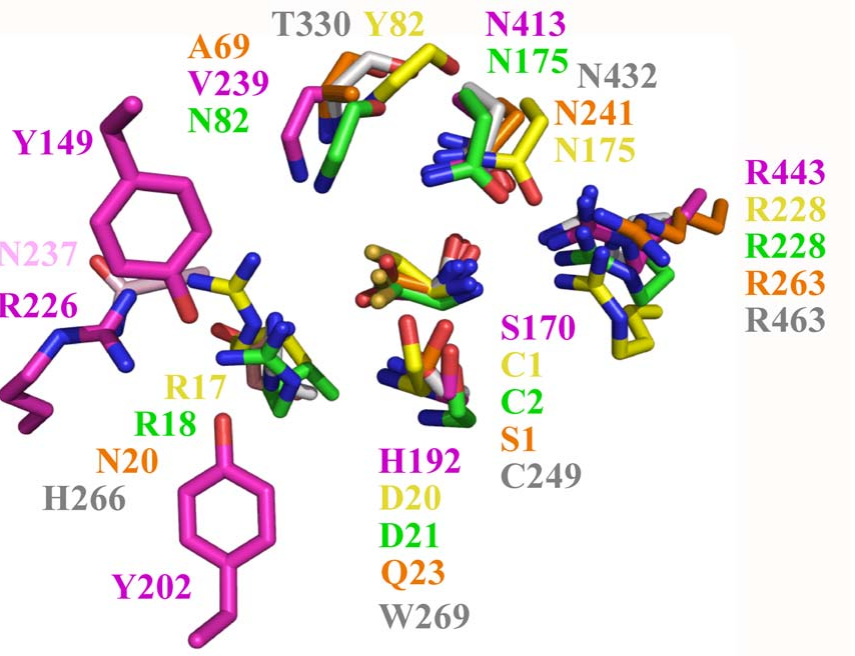
**Superposition of the conserved active site residues of the 66.3 kDa protein and the four most related N-terminal nucleophile hydrolases**. The conserved N-terminal nucleophile is shown completely, while of the ubiquitous asparagine and arginine residues as well as of the residue in the lower right corner only the side chains are represented, since the main chain atoms are not directly involved in catalysis. In contrast, of the other three residues, only the main chain atoms are depicted due to their participation in the catalytic reaction and a lack of sequence conservation. The residues are coloured by atom. Nitrogen, oxygen and sulphur atoms are shown in blue, red and light orange, respectively, for all structures, whereas the carbon atoms are represented distinctly for the various structures as follows: 66.3 kDa protein in grey (3FGR), cephalosporin acylase (1OQZ) in pink, penicillin V acylase (3PVA) in yellow, conjugated bile acid hydrolase (2BJF) in green, penicillin G acylase (1K5S) in orange.

Figure 1a, 2, 3, 4, 5 and 6 were prepared with PyMOL [36]. The simulated annealing omit maps of Figure 3 were calculated with CNS [31,32]. Additional file 1: Figure S1 and Additional file 2: Figure S2 were prepared with standard graphics programs, whereas Additional file 3: Figure S3 was prepared with CCP4 MOLECULAR GRAPHICS [37]. Additional file 4: Table S1 was produced in a text editing program. Additional file 5: Figure S4 and Additional file 6: Figure S5 were prepared with PyMOL [36] and CCP4 MOLECULAR GRAPHICS [37], respectively. CHEMSKETCH [38] was used for the generation of Additional file 7: Figure S6.

## Results and discussion

### Structure determination

The glycosylated lysosomal 66.3 kDa protein from mouse was produced and purified as described [17]. Two crystal forms were obtained under acidic conditions close to the physiological pH of the lysosomal compartment. The crystal form II was obtained under slightly different conditions concerning the composition of the protein and the reservoir solution. Both crystal forms belong to space group C2 and contain one molecule in the asymmetric unit but they differ in their cell parameters c and β. The 2.4 Å structure of the 66.3 kDa protein, which includes the residues 63–238 and 249–592 (PDB-ID: 3FBX), was previously obtained by means of sulphur SAD phasing [25] and revealed that the 28 kDa N-terminal and the 40 kDa C-terminal fragments of the processed 66.3 kDa protein still form one globular entity. The crystal structure refined to a resolution of 1.80 Å using another data set collected on the same crystal allowed to place four additional residues in the intermediate protein region between the two fragments, namely G245-S248 (PDB-ID 3FGR: xe1h; cleaved form) that turned out to be functionally important.

In the course of solving the crystallographic phase problem, further data sets were collected which turned out to be of interest as they represent different states of the maturation process of the 66.3 kDa protein. Diffraction data from a native crystal with a resolution limit of 2.4 Å (3FGT: native; cleaved) and from a non-isomorphous, potassium iodide soaked crystal (3FGW: KI; uncleaved) were analyzed in detail. The crystal structures described here were solved by means of Molecular Replacement using the initial structure of the 66.3 kDa protein (3FBX). While the protein monomers are arranged in a head-to-tail like manner in crystal form I (3FGR, 3FGT), two symmetry equivalent molecules form contacts head-to-head with each other in crystal form II (3FGW). Data collection and refinement statistics are summarized in Table 1.

The final structure 3FGR with the highest resolution of the three described structures, contains 180 residues of the N-terminal 28 kDa fragment and 344 residues of the C-terminal 40 kDa fragment (V63-T238 and G245-S248 in chain A, C249-P592 in chain B) (Table 1; Figure 1 and 2, Additional file 1: Figure S1. Schematic representation of the amino acid residue ranges comprised by the structures 3FGR, 3FGT and 3FGW). The N-terminal amino acids L47-P62, N239-L244 as well as the last C-terminal residues (W593, D594 and the eleven residues of the C-terminal affinity tag) are disordered in the structure 3FGR. However, it comprises the residues G245-S248, which could not be built in the initial structure (3FBX). By means of mass spectrometry, the residue range L47-S246/S248 has been shown to be present in the 28 kDa fragment as discussed in detail further below (Additional file 2: Figure S2. Mass spectrometry based analysis of the C-terminus of the 28 kDa fragment). The average temperature factors of only 24.1 Å^2 ^and 19.5 Å^2 ^for the amino acids of chains A and B, respectively, indicate an overall well defined conformation of the 66.3 kDa protein structure.

Non-interpreted electron density was found at the sulfhydryl group of C249, which is the N-terminal cysteine of the 40 kDa fragment. This sulfhydryl group appears to be partially oxidized (Figure 3) which could be a consequence of the fact that the 66.3 kDa protein was purified and crystallized in the absence of a reducing agent. C249 was modeled as cysteine sulfonic acid (OCS). The oxidized side chain of this N-terminal cysteine is involved in the octahedral coordination of a cation, which is additionally bound by the side chains of S246, E328, T330 and Y379 as well as by the main chain carbonyl group of D315. So far, the nature of this metal ion is not known. Since sodium acetate was present in the crystallization buffer and due to the absence of a peak in the anomalous electron density maps calculated with diffraction data sets collected at a wavelength of 0.8 Å, 1.7 Å and 1.9 Å as well as the results of fluorescence scans (carried out at BESSY BL14.1, data not shown), it seems likely that a Na^+ ^cation is bound to the protein. This is further supported by the octahedral coordination and metal-ligand atom distances of 2.7 – 3.1 Å [39].

In contrast to the structure 3FGR, in 3FGW, the amino acids G245-S248 are directly connected to C249 and the sulfhydryl group of C249 is not oxidized. (Table 1; Figure 3, see also Additional file 1: Figure S1. Schematic representation of the amino acid residue ranges comprised by the structures 3FGR, 3FGT and 3FGW). The coordination of a sodium cation at a position equivalent to 3FGR involves the same amino acids except for the replacement of OCS249 and S246 with only a single ligand, namely the main chain carbonyl group of G314. As outlined for the structure 3FGR, the nature of the metal cation is not known but it is assumed to be a Na^+ ^ion.

The structure 3FGT contains the residues D60-N239 of the N-terminal 28 kDa fragment and the residues C249-P592 of the C-terminal 40 kDa fragment (Table 1; see also Additional file 1: Figure S1. Schematic representation of the amino acid residue ranges comprised by the structures 3FGR, 3FGT and 3FGW). As observed in the high resolution structure, the N-terminal cysteine 249 of chain B of the structure 3FGT seems to be partially oxidized. Likewise, the same residues as in 3FGR except for the main chain carbonyl of G314 substituting S246 are involved in the coordination of the putative Na^+ ^ion (coordination sphere ≤ 3.7 Å).

### Overall structure

The structures 3FGR and 3FGT contain the cleaved form of the 66.3 kDa protein, which comprises two polypeptide chains corresponding to the 28 kDa and 40 kDa proteolytic fragments (Figure 2). If not stated otherwise, the structure 3FGR is described in detail below, since it has been refined at the highest resolution.

The compact globular structure shows two closely associated polypeptide chains (Figure 1 and 2) forming 37 hydrogen bonds as well as two salt bridges (K280-E127, R283-D107) (3FGT). The existence of the 28 kDa and 40 kDa chains as one entity is in accordance with the observation that both fragments as well as the uncleaved 66.3 kDa protein elute in a single 280 nm absorption peak from the affinity column, anion exchange column and gel filtration column during protein purification, respectively. The gel filtration peak corresponds to an apparent molecular weight of about 140 kDa indicating the existence of the 66.3 kDa protein as a stable dimer in solution. Contact areas between symmetry equivalent molecules in the crystals were analyzed with PISA [40]. In accordance with the results from the gel filtration, the calculated complexation significance score suggests the existence of a stable homodimer.

The N-terminal 28 kDa fragment consists of six α-helices (α1–α6) and four β-strands (β1–β4). The 40 kDa C-terminal fragment contains 13 β-strands (β5–β17), seven α-helices (α7–α13) as well as six 3/10-helices (η1–η6). Both fragments together form an αββα fold. The core is dominated by two highly twisted β-sheets. The six-stranded β-sheet (β-sheet I) is packed tightly against an extended eleven-stranded β-sheet (β-sheet II) (Figure 1a and 1b). The α- and 3/10-helices form two layers (α-layer I and II) that flank the central β-sheets on both sides engulfing them like a horseshoe and thus leaving one side of the β-sheet solvent accessible.

Most strands of the stacked β-sheets forming the central core derive from the 40 kDa fragment (β5–β17). They are slightly tilted against each other with β-strands β5, β6, β14–β17 forming β-sheet I with the topology β14–β5–β6–β15–β16–β17 and β7–β13 in combination with β1–β4 of the 28 kDa fragment that build β-sheet II with the topology β2–β1–β3–β4–β7–β8–β9–β10–β11–β12–β13 (Figure 1). All β-strands are oriented in an anti-parallel fashion except for a break at β7, which is oriented parallel to the preceding β4. The β-strands β1 and β2 partially protrude from the globular structure. Stabilization is achieved by some additional hydrophobic interactions, which are mainly formed between the α-helices α4 and α9 and β-strands β4 and β7. Additionally, two intramolecular disulfide bridges are formed between C147 and C157 of the N- as well as between C497 and C500 of the C-terminal fragment (Figure 2). In contrast, intermolecular disulfide bonds are not observed which is in accordance with the electrophoretic separation of the fragments under non-reducing conditions [17].

The crystal structure contains seven N-acetylglucosamine moieties (NAG) in total, which are part of five N-glycans at the asparagine residues 93, 115, 236, 441 and 520 (Figure 2) and are well defined in the electron density map. The glycosylation sites are evenly distributed on the surface of the molecule. The three N-glycosylation sites of the 40 kDa fragment surround a prominent cavity – the putative substrate binding pocket – in close proximity, while the remaining two sites are localized on the opposing side of the protein molecule (Figure 2).

### Differences between the three structures of the 66.3 kDa protein

Superposition of the three refined structures of the 66.3 kDa protein reveals only slight variations in the overall conformation. The r.m.s. deviations between the structures 3FGT and 3FGW compared to 3FGR amount to 0.36 Å and 0.35 Å for 520 common Cα atoms (V63-T238 and C249-P592), respectively. The most significant difference concerns the peptide bond connecting residues S248 and C249. While in 3FGR and 3FGT there is no covalent bond between S248 and C249, continuous electron density was observed between these residues in 3FGW indicating the uncleaved form of the protein (Figure 3). Upon cleavage, the conformation of S248 and C249 changes significantly. The incision causes a rearrangement of S248 leading to an extensive hydrogen bonding network which includes a salt bridge formed between the terminal carboxyl group of S248 and the side chain of R531 (3FGR).

In the uncleaved structure (3FGW), C249 falls into the disallowed region of the Ramachandran plot and exhibits *cis *configuration, while after cleavage it is *trans *and located in the core region of the Ramachandran plot corresponding to β-strand conformation. In analogy to other auto-proteolytically cleaved enzymes [41], this strong distortion most likely helps in providing the potential required for the proteolytic cleavage (see below). The cleavage is additionally accompanied by slight changes of the torsion angles of the adjacent residue S248, which is within the allowed region of a left-handed α-helix before and in the core β-strand region of the Ramachandran plot after the cleavage (Figure 3).

The proximate residues N239/T240 – L244 of the linker peptide are flexible in all three structures. Due to significant radiation damage occurring during data collection, the crystals were not suitable for further experiments. However, mass spectrometric analysis (MS) was performed with purified 66.3 kDa protein incubated under crystallization conditions. This experiment unambiguously showed that the residues N239/T240 – L244 are present in the 28 kDa fragment of the cleaved protein forms represented by 3FGR and 3FGT as follows (Additional file 2: Figure S2. Mass spectrometry based analysis of the C-terminus of the 28 k Da fragment).

In order to determine the exact C-terminus of the 28 kDa fragment derived from processed 66.3 kDa protein, purified 66.3 kDa protein was incubated under crystallization conditions (3FGT) with N-Gylcosidase F (PNGase). PNGase cleaves all types of asparagine linked N-glycans and thus transforms the respective asparagine into aspartate residues within glycosylated proteins upon complete deglycosylation [42]. Additional file 2: Figure S2a shows the separation of the purified 66.3 kDa protein on a 1D SDS-PAGE before (lane 1) and after PNGase treatment (lane 2). MS after in-gel digestion of the Coomassie stained peptides showed that band 1 contains the full length protein, while band 2 represents the processed 40 kDa fragment (of note, close inspection of this particular Coomassie band revealed a doublet) and band 3 the processed 28 kDa fragment. The fuzzy staining of protein and its fragment is caused by the glycosylation on various asparagine residues [17]. After PNGase treatment the corresponding bands are much sharper, and indeed the processed 40 kDa fragment appears as doublet (bands 4 and 5). Band 6 contains the 28 kDa fragment. To detect the C-terminus of the latter fragment by MS, we manually inspected the generated MS and MS/MS spectra for peptides with the calculated mass (MW_cal_) of the C-terminal tryptic peptide (TNTKPSLGSGS, MW_cal _= 1047.5196) or for C-terminal tryptic peptides that lack one or more C-terminal amino acids. Figure S2 shows annotated MS (panels A and B, small inserts) and MS/MS spectra from a peptide found in the MS analysis that encompasses the intact C-terminus TNTKPSLGSGS (238–248, Figure S2b) and a shorter peptide with the sequence TNTKPSLGS (238–246, Figure S2c) found in the same analysis (Additional file 2: Figure S2. Mass spectrometry based analysis of the C-terminus of the 28 kDa fragment). The MS/MS spectra clearly show a y-type ion series that unambiguously reveals the sequence of the peptides. The mass deviation of the calculated and experimentally determined mass of both peptides is ≤ 2 ppm. However we could not identify a peptide which only lacks one C-terminal residue (S248), i.e. TNTKPSLGSG (238–247). Furthermore, we could not monitor any fragments shorter than the truncated one. In summary, the MS analysis proofs that under crystallization conditions the processed 28 kDa fragment comprises residues L47-S248 (....TNTKPSLGSGS) and also occurs as a slightly shorter form truncated at the C-terminus by two amino acid residues, i.e. containing the residues L47-S246 (....TNTKPSLGS).

Based on these observations, we assume that the processing step which gives rise to the 28 kDa and 40 kDa fragments starts with a cleavage between S248 and C249. According to the mass spectrometric results, the 28 kDa fragment which is derived after the proteolytic cleavage between S248 and C249 occurs in two species represented by the structures 3FGR (L47-S248) and 3FGT (L47-S246), respectively. Due to the absence of the last two C-terminal residues G247 and S248 in the shorter version of the 28 kDa fragment, the linker peptide probably cannot interact with the side chain of R531 anymore. Thus, we assume the structure 3FGT to represent the shorter version of the 28 kDa fragment including L47-S246 with the residues T240-S248 completely disordered.

The amino acids G245 – S248 exhibit an ordered conformation in the structures 3FGR and 3FGW (Figure 4). Interestingly, the loop residues G245-S248 adopt quite different conformations in 3FGR and 3FGW (Figure 4, Additional file 3: Figure S3. Comparison of the solvent accessibility of the putative substrate binding pocket in the three structures). In 3FGR the residues G245 – S248 are oriented perpendicular to the first β-strand of the 40 kDa fragment (β5), whereas they extend this β-strand in 3FGW, even though the β-strand secondary structure is significantly distorted. Due to the disorder of the residues T240-S248 in 3FGT a large pocket with a highly negative surface potential becomes solvent accessible (Figure 4 and Additional file 3: Figure S3. Comparison of the solvent accessibility of the putative substrate binding pocket in the three structures). This cavity emerged to have a putative important role for the function of the 66.3 kDa protein as is described below.

### Structurally related proteins

In order to obtain insight into the function of the lysosomal 66.3 kDa protein, the Protein Data Bank (PDB) was searched for structurally related proteins with known function. The retrieval using the program DALI [43] revealed significant similarities to cephalosporin acylase (CA) [44] (Figure 5), two different kinds of penicillin acylase (penicillin acylase G (PGA) [45] and V (PVA) [46]), as well as conjugated bile acid hydrolase (CBAH) [47] (Table 2). For these four bacterial proteins the number of the structurally equivalent residues is in the range from 222 (PVA) to 360 (CA) with regard to 520 amino acids of the 66.3 kDa protein. The r.m.s. deviations for the positions of aligned Cα atoms amount to 3.0 Å (PVA) – 3.6 Å (CA). Furthermore, some less similarity was found to inosine monophosphate (IMP) cyclohydrolase (IMPC) [48] and proteasome subunits [49,50] (for details see Additional file 4: Table S1. Extended list of structures with a similar fold as the 66.3 kDa protein revealed using the program DALI). Interestingly, only a few of the aligned residues are conserved between the 66.3 kDa protein and the structurally related proteins. Merely 6% (PVA, CBAH) to 14% (IMPC) of the structurally equivalent amino acids are identical. Superpositions of the 66.3 kDa protein with CA and CBAH as representives are shown in Figure 5 and in Additional file 5: Figure S4. Superposition of linker residues and ligands of the 66.3 kDa protein, cephalosporin acylase (CA) and conjugated bile acid hydrolase (CBAH). All structures exhibit the akin central overall fold with the highest degree of similarity concerning the β-sheet core, while the arrangement of the surrounding α-helices differs.

**Table 2:** Comparison of the 66.3 kDa protein with Ntn hydrolases of known structure (DALI).

**protein**	**Abbreviation**	PDB-ID*	**Z-score**	**rmsd** **[Å]**	**L_ali_**	**N_res_**	**% ID**
cephalosporin acylase	CA	1oqz	17.0	3.6	360	684	11

penicillin V acylase	PVA	3pva	16.2	3.0	222	334	6

conjugated bile acid (= choloylglycine) hydrolase	CBAH	2bjf	16.2	3.1	224	328	6

penicillin G acylase	PGA	1k5s	15.4	3.4	244	557	11

IMP cyclohydrolase	IMPC	2ntm	8.4	3.2	165	202	14

20 S proteasome	-	1ryp	8.3	3.1	161	205	7

Only hits with a Z-score ≥ 7 and with an assigned cellular function are listed here. A complete list of all revealed similar structures can be found in Additional file 4: Table S1. Extended list of structures with a similar fold as the 66.3 kDa protein revealed using the program DALI.

* For redundant protein, the PDB-ID and the corresponding values are given only for the best hit. Z-score = value for comparison. Hits with Z-scores ≤ 2 are spurious. rmsd = root mean square deviation between the aligned residues, L_ali_: number of structurally equivalent residues, N_res_: number of amino acids in the protein, % ID: percentage of identical amino acids over all structurally equivalent residues.

Although most of the acylases lack significant sequence similarity among each other, they belong to a single superfamily termed Ntn hydrolase, which is defined by a common fold. The characteristic structural motif is a four-layered αββα sandwich [51,52] (Figure 1). Based on the crystal structure, the 66.3 kDa protein could be assigned to this superfamily.

The PDB contains the crystal structure of another lysosomal Ntn hydrolase, namely that of aspartylglucosaminidase (AGA) [53]. However, this enzyme has not been revealed by DALI, and secondary structure matching for the C-terminal fragment only allowed the alignment of 80 residues with r.m.s. deviations of 4.1 Å.

### Putative active site

Based on structural homology, the lysosomal 66.3 kDa protein belongs to the superfamily of Ntn hydrolases. All functional Ntn hydrolases known so far are activated by autocatalytic cleavage. The N-terminal residue generated at the cleavage site represents the canonical catalytic residue and performs a nucleophilic attack on the carbonyl carbon of the non-peptide amide bond of the substrate. The catalytically essential nucleophile is either threonine, serine or cysteine (such as serine 170 of CA, serine β1 of PGA, threonine 206 of lysosomal AGA and cysteine 2 of CBAH and cysteine 1 of PVA). While the hydroxyl oxygen or the sulphur atom of the N-terminal residue acts as the nucleophile, its free α-amino group serves as the general base. Based on the superposition of the 66.3 kDa protein with known Ntn hydrolases (Figure 5 and 6, see also Additional file 6: Figure S5. Surface representation of the substrate binding pocket of the 66.3 kDa protein according to its hydrophilic/hydrophobic character), we suggest C249 at the N-terminus of the 40 kDa fragment to represent the conserved nucleophilic residue. C249 becomes solvent accessible only after the proteolytic cleavage between S248 and C249 and as soon as the C-terminus of the N-terminally located linker peptide is trimmed and thus becomes flexible probably moving to the surface of the protein as can be seen by comparison of the structures 3FGR and 3FGT (Figure 3 and Additional file 3: Figure S3. Comparison of the solvent accessibility of the putative substrate binding pocket in the three structures).

In addition, other known active site residues of Ntn hydrolases are conserved like an asparagine and an arginine residue (Figure 6). These residues corresponding to N432 and R463 of the 66.3 kDa protein have been shown to be essential in other Ntn hydrolases, e.g. for the catalytic activity of PGA (N241 and R263) [54,55]. In 3FGR, the Od atom of the asparagine is hydrogen-bonded to the amino group of the N-terminal nucleophilic amino acid as well as to the side chain of the arginine as observed in all four Ntn hydrolase structures closely related to the 66.3 kDa protein (Figure 6). The Nd of the asparagine forms hydrogen bonds with both a backbone carbonyl oxygen of a residue located nearby (T330) and – in the crystal structures 3FGR and 3FGT – with the sulfonic acid side chain of the oxidized N-terminal cysteine 249.

Another residue conserved in the active site of Ntn hydrolases is either a histidine or an arginine corresponding to H266 of the 66.3 kDa protein (Figure 6). A histidine occupies this position in some Ntn hydrolases which exhibit an N-terminal cysteine as the nucleophilic residue like the 66.3 kDa protein such as glutamine phosphoribosylpyrophosphate (PRPP) amidotransferase [56] and glucosamine 6-phosphate synthase [57,58]. Due to the acidic lysosomal environment H266 is protonated and therefore able to take over the role of the arginine. The positively charged histidine side chain most likely enhances the nucleophilic character of the catalytic N-terminal amino acid by decreasing its pK_a _value. Thus, the histidine/arginine conservation is most likely based rather on the catalytic mechanism than on substrate specificity.

The backbone nitrogen of T330 and Nd2 of N432 most likely form the oxyanion hole in the 66.3 kDa protein. A third residue appears to be involved as well, namely W269. Like the structural equivalents, the backbone nitrogen of W269 forms a hydrogen bond with the N-terminal nucleophile. The corresponding residues Qβ23 of PGA and H192 of CA form a second hydrogen bond to the N-terminal amino group or Od of the conserved active site asparagine, respectively, via their side chains. Mutation of H192 to serine completely abolished autoproteolysis showing this residue to have an important role not only for the catalytic turnover of a substrate, but also for the activation of CA. W269 is not able to form equivalent interactions. Based on this difference, we suggest W269 to be important for the catalytic activity but not essential for its autoproteolytic activation.

Thus, all active site residues as well as characteristic hydrogen bonding patterns of the Ntn hydrolases CBAH, CA, PVA and PGA are conserved in the 66.3 kDa protein (Figure 6) suggesting that the same reaction mechanism is applied to hydrolyze a non-peptide amide bond. In contrast, several amino acids involved in substrate binding do not have functional equivalents, but this lack of sequence conservation concerning the binding site is not surprising and has been observed for almost all members of the Ntn hydrolase superfamily [52]. It reflects the wide variety of substrate molecules despite the similar active site structure. Polar side chains in proximity to the catalytic center suitable for interactions with a putative substrate molecule are delivered by S225, T238, N274 and T378 of the 66.3 kDa protein.

### Putative substrates

So far, the substrates of the 66.3 kDa protein remain unknown. The members of the Ntn hydrolase superfamily differ significantly in substrate specificity and in the respective substrate binding pocket. However, the structural classification of the 66.3 kDa protein as an Ntn hydrolase implies a hydrolytic activity on a kind of non-peptide amide bond as commonly observed for Ntn hydrolases. Based on the high similarity to members of the choloylglycine hydrolase family (CBAH and PVA), the 66.3 kDa protein might have an enzymatic function related to that of other lysosomal members of this family such as acid ceramidase (AC) and the NAE-hydrolyzing acid amidase (NAAA). According to this hypothesis, the 66.3 kDa protein could be involved in the degradation of N-acylethanolamines (NAEs) of specific chain lengths leading to 2-aminoethanol (ethanolamine) and the corresponding free fatty acids.

NAEs represent a class of tissue hormones (mediators) that are synthesized in a variety of organisms and tissues [59] (reviewed in [8,60,61]). In mammalia, NAEs normally occur in trace amounts, but under pathological conditions tissue NAE levels increase significantly [8,62,63]. Anti-inflammatory [64-66], neuroprotective [67], immunosuppressive [68] and analgesic [9] functions have been determined for various NAEs. Thus, their spread has to be strictly regulated.

The choloylglycine hydrolase NAAA is involved in the degradation of NAEs in lysosomes [69] (reviewed in [70,71]). In contrast, the two further known lysosomal members of this family, aspartylglucosaminidase (AGA, see above) and acid ceramidase (AC) [71,72] hydrolyse the N-glycosidic bond between oligosaccharides and asparagines and act on the amide bond of ceramides, respectively.

The best substrate of NAAA, which shows optimal activity at acidic pH, is N-palmitoyl-EA. A second NAE-degrading enzyme specific for a different set of NAEs differing in chain length and particularly in the saturation status of the fatty acid moieties is the fatty acid amide hydrolase (FAAH) [73,74]. This membrane-bound enzyme of the ER and/or Golgi compartment is most active at neutral pH [75-77]. In contrast to NAAA, FAAH does not belong to the Ntn hydrolase superfamily, but to the amidase signature family.

However, enzyme(s) degrading all other kinds of NAEs such as N-stearoyl- (C18:0), N-γ-linolenoyl- (C18:3), and some longer fatty acid EAs (C22:1, C22:6) have not been identified so far. Hence, the 66.3 kDa protein could be involved in the hydrolysis of one or several of these compounds.

### Activation by auto-proteolytic removal of the linker peptide

Activation of Ntn hydrolases requires an auto-proteolytic cleavage resulting in the removal of several amino acids or even a whole polypeptide chain N-terminal of the nucleophilic residue. CA which exhibits the most significant structural similarity to the 66.3 kDa protein is activated by a multi-step maturation process leading to a two chain form of the protein [78]. During this maturation, two proteolytic cleavages cause the release of a spacer peptide, which makes the substrate binding pocket solvent accessible. The lysosomal 66.3 kDa protein bears such a highly flexible linker region most likely comprising the amino acids N239 to S248, which connect the 28 kDa fragment and the 40 kDa fragment prior to maturation (Figure 2 and 3, Additional file 3: Figure S3. Comparison of the solvent accessibility of the putative substrate binding pocket in the three structures).

Most known Ntn hydrolases [44,79] as well as inteins [80] contain a glycine residue adjacent to the nucleophilic amino acid on the N-terminal side. However, in the 66.3 kDa protein, a serine residue (S248) is located at the equivalent position and similar exceptions have been found in the lysosomal Ntn hydrolase AGA (D182) [81] as well as in plant asparaginases [82]. However, in the 66.3 kDa protein, a glycine residue is located two amino acids apart from the catalytic C249 with a serine residue in between. N-terminal of this glycine 247 another glycine-serine pair (G245, S246) probably further increases the flexibility of the linker peptide. In the structure 3FGW, the linker residue range from G245 to S248, which is still covalently bound to C249, exhibits a strongly distorted conformation with the scissile peptide bond between S248 and C249 in *cis *conformation. Upon the first proteolytic cleavage (see Additional file 7: Figure S6. Putative mechanism of the auto-proteolytic cleavage between S248 and C249 during the maturation process of the 66.3 kDa protein), the strained conformation is released, as becomes obvious in the structure 3FGR (Figure 3), in which all peptide bonds of the defined part of the linker exhibit *trans *conformation. These results are in agreement with similar observations regarding the autoproteolytic activation process of lysosomal AGA.

For CA, a second autocatalytic cleavage releasing a spacer peptide has been reported that requires E159 [78,83]. The superposition of CA and the 66.3 kDa protein shows the side chain carboxyl groups of E159 and E153, respectively, to be located similarly. However, they belong to non-equivalent β-strands, and a residue feasible to form the oxyanion hole for a putative second autoproteolytic cleavage between T238 and N239 in the 66.3 kDa protein could not be identified. Upon cleavage between S248 and C249, the C-terminal residues probably protrude from the protein making them accessible for successive removal. Thus, we suggest the C-terminus of the 28 kDa fragment (from residue S248) to be trimmed by proteases which are quite abundant in the lysosomal compartment rather than to be released by a second autocatalytic step. *In vivo*, the N-glycan attached to N236, which was shown to be included in the mature 28 kDa fragment [17], should protect the 28 kDa fragment against further C-terminal degradation. The crystallized protein had not reached the lysosomal compartment due to a capacity overload of the MPR-mediated transport system, but was secreted by exocytosis as a precursor. Therefore, the requirement of lysosomal enzymes for the later steps of maturation as reported for the lysosomal Ntn hydrolase AGA [41,53,84] are also in agreement with the presence of amino acid residues C-terminal of the glycosylated N236 in the crystal structures (Figure 3). By means of mass spectrometric analysis of the purified 66.3 kDa protein, S248 and S246, respectively, have been identified as the C-terminal residue of two occurring variations of the 28 kDa fragment. The exact length of the linker might not have any effect on the acylase activity as reported for CA from different *Pseudomonas *species for which variations from 8 to 11 amino acids occur [83,85-89]. However, most likely full access to the putative catalytic site arranged around C249 as observed in the structure 3FGT is only provided after trimming of the C-terminus of the 28 kDa fragment.

## Conclusion

Three crystal structures of the lysosomal 66.3 kDa protein from mouse were determined (PDB-ID 3FGR, 3FGT, 3FGW) representing different states of its post-translational processing that gives rise to a 28 kDa N- and a 40 kDa C-terminal fragment. The structures shed light on this maturation procedure, which includes an autocatalytic cleavage. Additionally, they provide initial insight into the so far unknown function of the 66.3 kDa protein.

The major difference between the three structures concerns a linker peptide of about ten amino acids N-terminal of C249. In the uncleaved 66.3 kDa protein form, S248 is still covalently connected to C249 (3FGW) and occupies a large cavity. During maturation, the peptide backbone is incised between S248 and C249 (3FGR). In the cleaved 66.3 kDa protein form 3FGR, S248 still occupies a large cavity. Subsequently, the linker region seems to become highly flexible due to further trimming of the C-terminus of the 28 kDa fragment by two residues and might move to the surface of the protein. Thus, a deep pocket becomes accessible for the binding of putative substrates (3FGT).

The structures of the 66.3 kDa protein reveal significant similarities to several bacterial acylases, which belong to the N-terminal nucleophile (Ntn) hydrolase superfamily. Based on this structural homology including both the overall fold and the active site residues, the 66.3 kDa protein could be assigned to the superfamily of Ntn hydrolases – a classification which could not have been derived from the amino acid sequence due to the lack of a respective homology.

Commonly, Ntn hydrolases act on non-peptide amide bonds. Thus, molecules exhibiting a non-peptide amid bond most likely serve as substrates of the 66.3 kDa protein. The potential target molecules comprise N-acylethanolamines (NAEs). The lysosomal compartment plays a major role in the regulation of the NAE level in the cell, but the degradation of the entire set of the various NAEs cannot be explained completely by the action of the enzymes NAAA and FAAH, which so far have been shown to be involved. Certainly, this hypothesis has to be confirmed by further biochemical studies. Currently, a gene trap knockout mouse is under construction and might help to evaluate the physiological function of the 66.3 kDa protein.

Alternatively, other non-peptide amide bonds seem to be suitable substrates of the 66.3 kDa protein. They occur only in few natural compounds such as lipid-anchored proteins, sphingosines and acetylated lysine residues, and the 66.3 kDa protein might be involved in their degradation. While enzymes responsible for the degradation of farnesylated and geranylated proteins or peptides arising from lipid-modified proteins have been identified, an activity for the demyristoylation of proteins within lysosomes is only speculative at present as reviewed in [90]. Acetylated lysine residues are beyond others found in the basic charged N-terminal region of histones [91-93], which have important roles in the organization of the DNA structure in eukaryotic cells and are crucial for the regulation of gene expression [94]. In contrast to the already characterized regulatory histone deacylases (HDACs) the 66.3 kDa protein might remove the acetyl moiety from the proteins in the course of protein degradation.

## Abbreviations

AC: acid ceramidase; AGA: aspartylglucosaminidase; CA: cephalosporin acylase; CBAH: conjugated bile acid (= choloylglycine) hydrolase; CID: collision induced dissociation; IMPC: inosine monophosphate cyclohydrolase; LIT: linear ion trap; LC: liquid chromatography; MPR: mannose 6-phosphate receptor; MS: mass spectrometry; MS/MS: coupled tandem mass spectrometry; NAAA: N-acylethanolamine hydrolyzing acid amidase; NAE: N-acylethanolamine; PGA: penicillin G acylase; PVA: penicillin V acylase; r.m.s.d: root mean square deviation; R_p.i.m_: precision-indicating R factor; R_merge_: merging R factor; TGN: trans-Golgi network.

## Authors' contributions

MK and TL overexpressed and purified the 66.3 kDa protein. KL performed a final purification step, the crystallization and the X-ray diffraction data collection. Crystal structure refinement and analysis were carried out by KL, AD and RF. Mass spectrometric analysis was performed by HU. All authors were involved in the preparation of the manuscript, and all authors read and approved the final manuscript.

## Supplementary Material

Additional file 1 **Figure S1**. Schematic representation of the amino acid residue ranges comprised by the structures 3FGR and 3FGT. The residues of the N-terminal 28 kDa fragment, the linker region and the C-terminal 40 kDa fragment, which are included in each structure, are represented as boxes coloured in yellow, light grey and blue, respectively. The first and the last residue of each region are given in bold letters. The dotted lines represent missing residues of the intermediate region. Click here for file

Additional file 2 **Figure S2. Mass spectrometry based analysis of the C-terminus of the 28kDa fragment**. (a) SDS-PAGE analysis of the purified 66.3 kDa protein after incubation under crystallization conditions (3FGT) prior to (lane 1) and after (lane 2) PNGase treatment. (b, c) Mass spectrometric chromatograms of the C-terminal peptide species of the 28 kDa fragment, that are present in the protein batch: T238-S248 (b) and T238-S246 (c). Click here for file

Additional file 3 **Figure S3**. Comparison of the solvent accessibility of the putative substrate binding pocket in the three structures. The residues P61/P60/V63-T238 (3FGW/3FGT/3FGR) of the N-terminal and C249-P592/D594 (3FGR+3FGT/3FGW) of the C-terminal fragment are shown as orange and blue surfaces. The residues N239-S248 are shown in stick mode (same colour code as in Figure 3), whereas the coordinated metal ion is represented by a black sphere. Click here for file

Additional file 4 **Table S1**. Extended list of structures with a similar fold as the 66.3 kDa protein revealed using the program DALI. Click here for file

Additional file 5 **Figure S4**. Superposition of linker residues and ligands of the 66.3 kDa protein, cephalosporin acylase (CA) and conjugated bile acid hydrolase (CBAH). The active site residues of the 66.3 kDa protein (3FGR) are represented according to Figure 6 with the carbon atoms coloured in light grey. The linker residues N239 as well as G245-S248 of the structures 3FGR and 3FGW are shown as black and blue stick model, respectively. They fit well with the linker regions and ligands of the aligned structures of CA and CBAH, which are coloured as follows: glutarate in yellow, 7-β-(4-carboxybutanamido)-cephalosporanic acid in light orange (1JVZ) [89], D161-G169 of CA in dark orange [44], taurine and deoxycholate in red [47]. Click here for file

Additional file 6 **Figure S5**. Surface representation of the substrate binding pocket of the 66.3 kDa protein according to its hydrophilic/hydrophobic character. The residues V63-T238 as well as C249-P592 of the structure 3FGR are shown in surface representation. Hydrophilic amino acids and glycans are coloured in yellow, whereas hydrophobic residues are shown in grey. The linker residues G245-S248 (3FGR) are shown in stick mode, the coordinated Na^+ ^ion is represented as a blue sphere. Click here for file

Additional file 7 **Figure S6**. Putative mechanism of the auto-proteolytic cleavage between S248 and C249 during the maturation process of the 66.3 kDa protein. Residues of and adjacent to the scissile peptide bond are labeled in blue, while residues of which side chain and backbone atoms are involved in the represented interactions, are labeled in black and grey, respectively. The first nucleophilic attack at the carbonyl carbon of S248 by the sulfhydryl group of C249 and the subsequent formation of the oxyanion are indicated by orange arrows. Possible attacks following this transition state are represented by green and blue arrows depending on whether the oxygen atom is part of the serine side chain or of a bound water molecule. Click here for file
