# Supplementary figures and images for: Initial insight into the function of the lysosomal 66.3 kDa protein from mouse by means of X-ray crystallography
Source: BMC Struct Biol. 2009 Aug 25;9:56. doi: 10.1186/1472-6807-9-56 (PMC2739207; doi:10.1186/1472-6807-9-56)

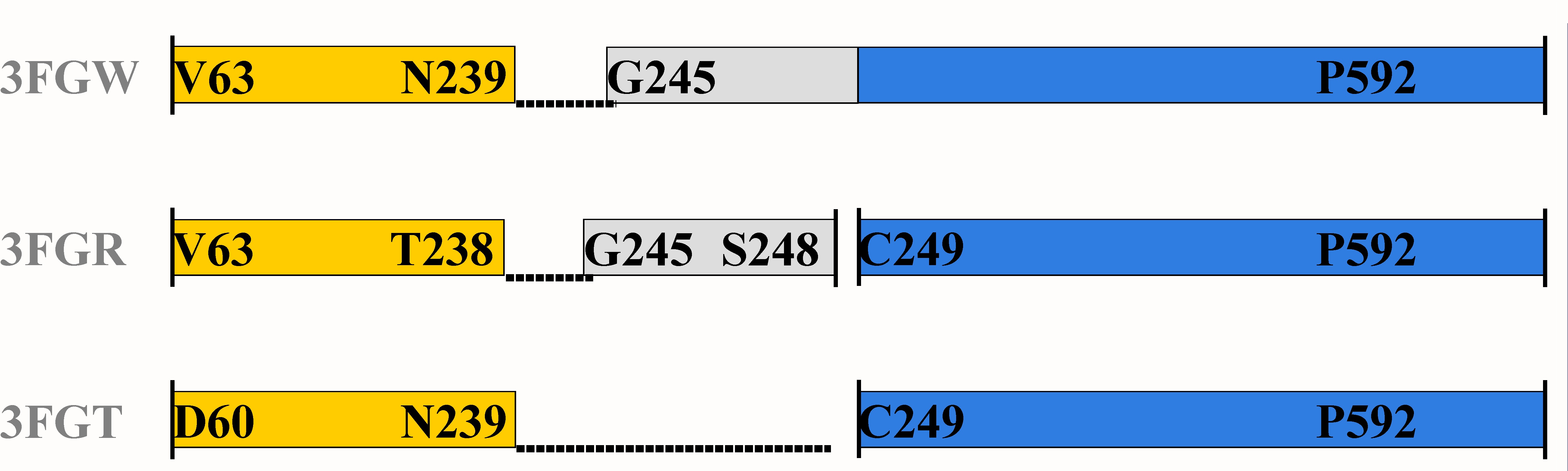

Supplement: Additional file 1 — Figure S1. Schematic representation of the amino acid residue ranges comprised by the structures 3FGR and 3FGT. The residues of the N-terminal 28 kDa fragment, the linker region and the C-terminal 40 kDa fragment, which are included in each structure, are represented as boxes coloured in yellow, light grey and blue, respectively. The first and the last residue of each region are given in bold letters. The dotted lines represent missing residues of the intermediate region. [file 1472-6807-9-56-S1.jpeg]

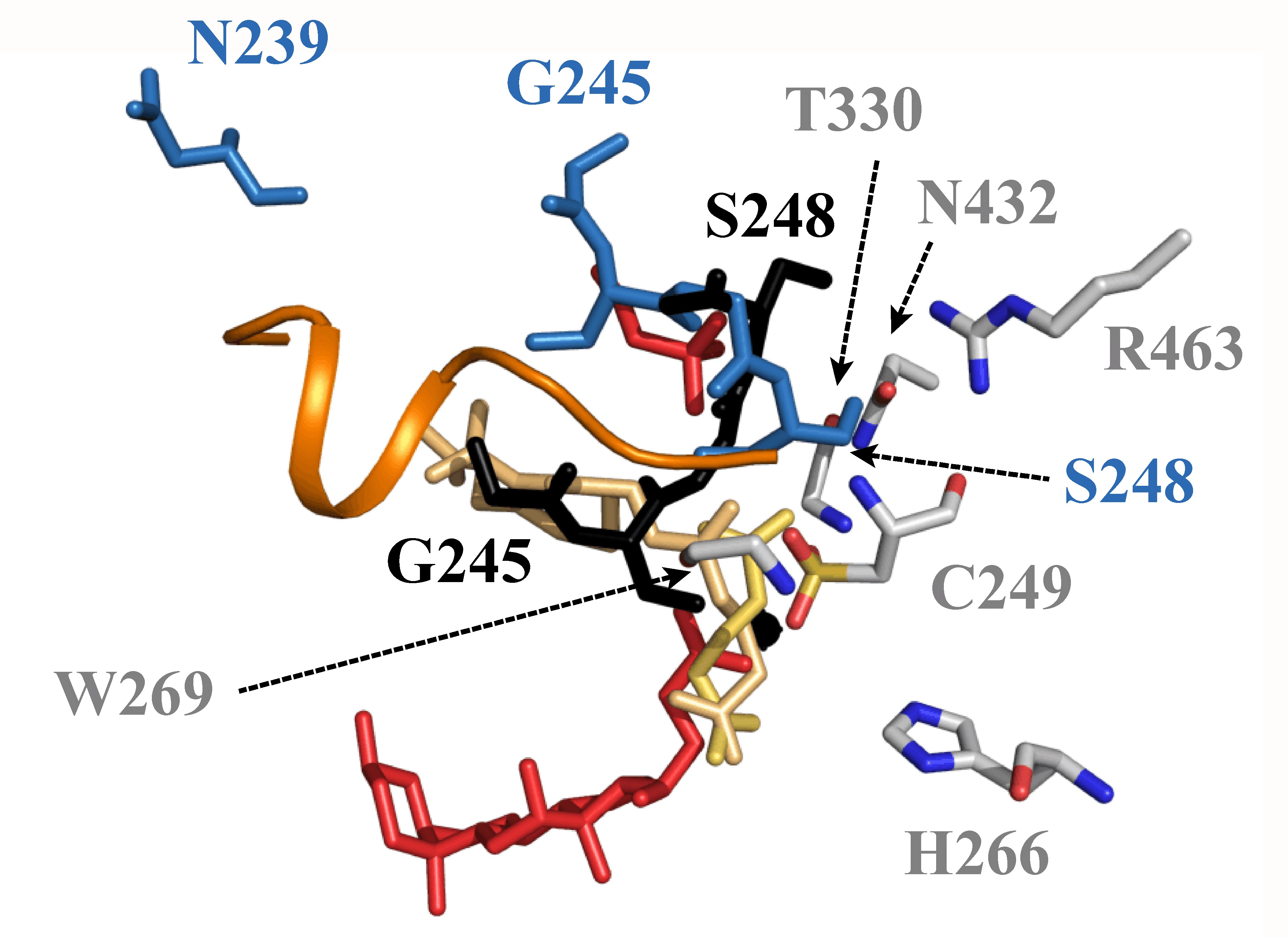

Supplement: Additional file 5 — Figure S4. Superposition of linker residues and ligands of the 66.3 kDa protein, cephalosporin acylase (CA) and conjugated bile acid hydrolase (CBAH). The active site residues of the 66.3 kDa protein (3FGR) are represented according to Figure 6 with the carbon atoms coloured in light grey. The linker residues N239 as well as G245-S248 of the structures 3FGR and 3FGW are shown as black and blue stick model, respectively. They fit well with the linker regions and ligands of the aligned structures of CA and CBAH, which are coloured as follows: glutarate in yellow, 7-β-(4-carboxybutanamido)-cephalosporanic acid in light orange (1JVZ) [89], D161-G169 of CA in dark orange [44], taurine and deoxycholate in red [47]. [file 1472-6807-9-56-S5.jpeg]

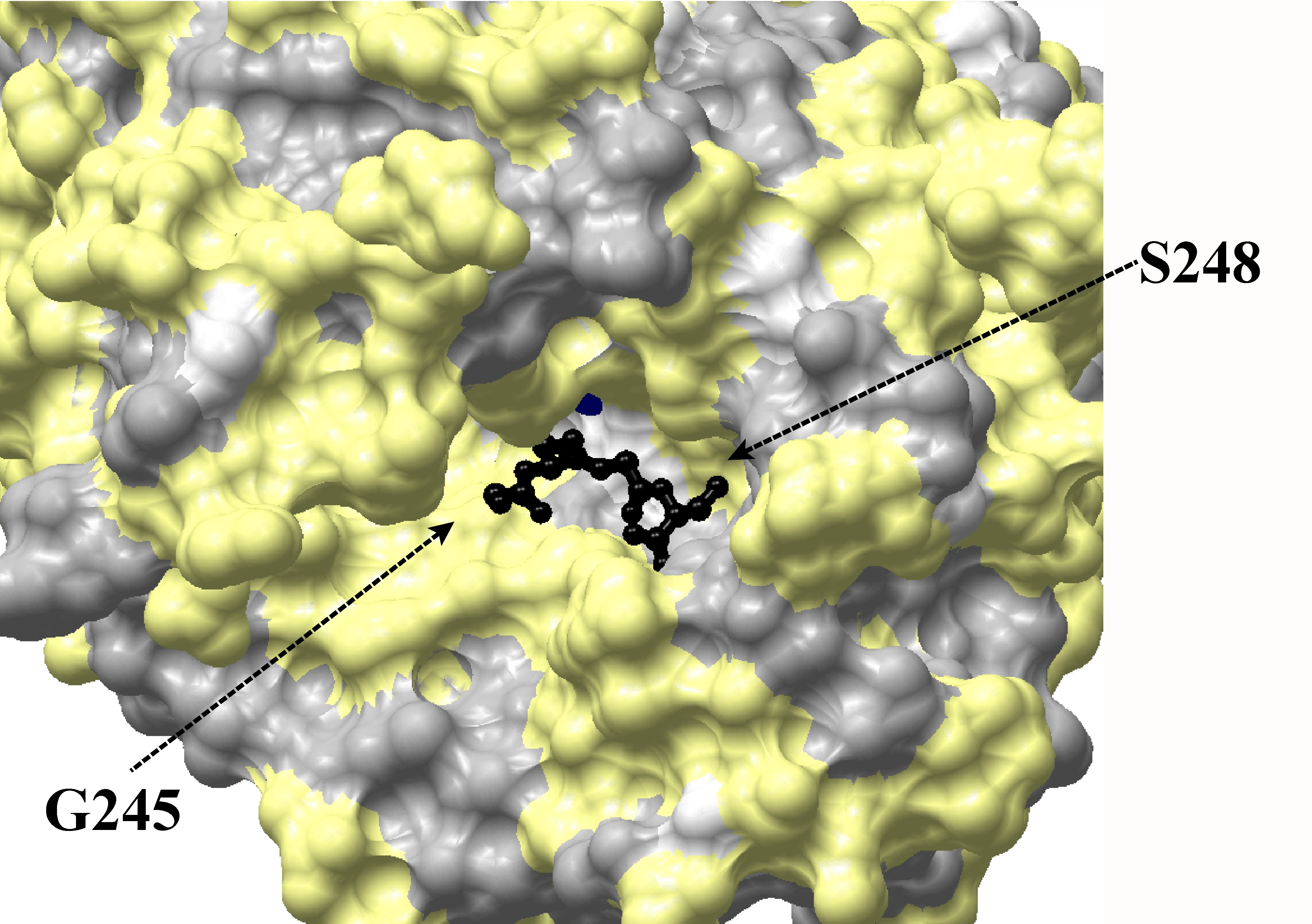

Supplement: Additional file 6 — Figure S5. Surface representation of the substrate binding pocket of the 66.3 kDa protein according to its hydrophilic/hydrophobic character. The residues V63-T238 as well as C249-P592 of the structure 3FGR are shown in surface representation. Hydrophilic amino acids and glycans are coloured in yellow, whereas hydrophobic residues are shown in grey. The linker residues G245-S248 (3FGR) are shown in stick mode, the coordinated Na+ ion is represented as a blue sphere. [file 1472-6807-9-56-S6.jpeg]

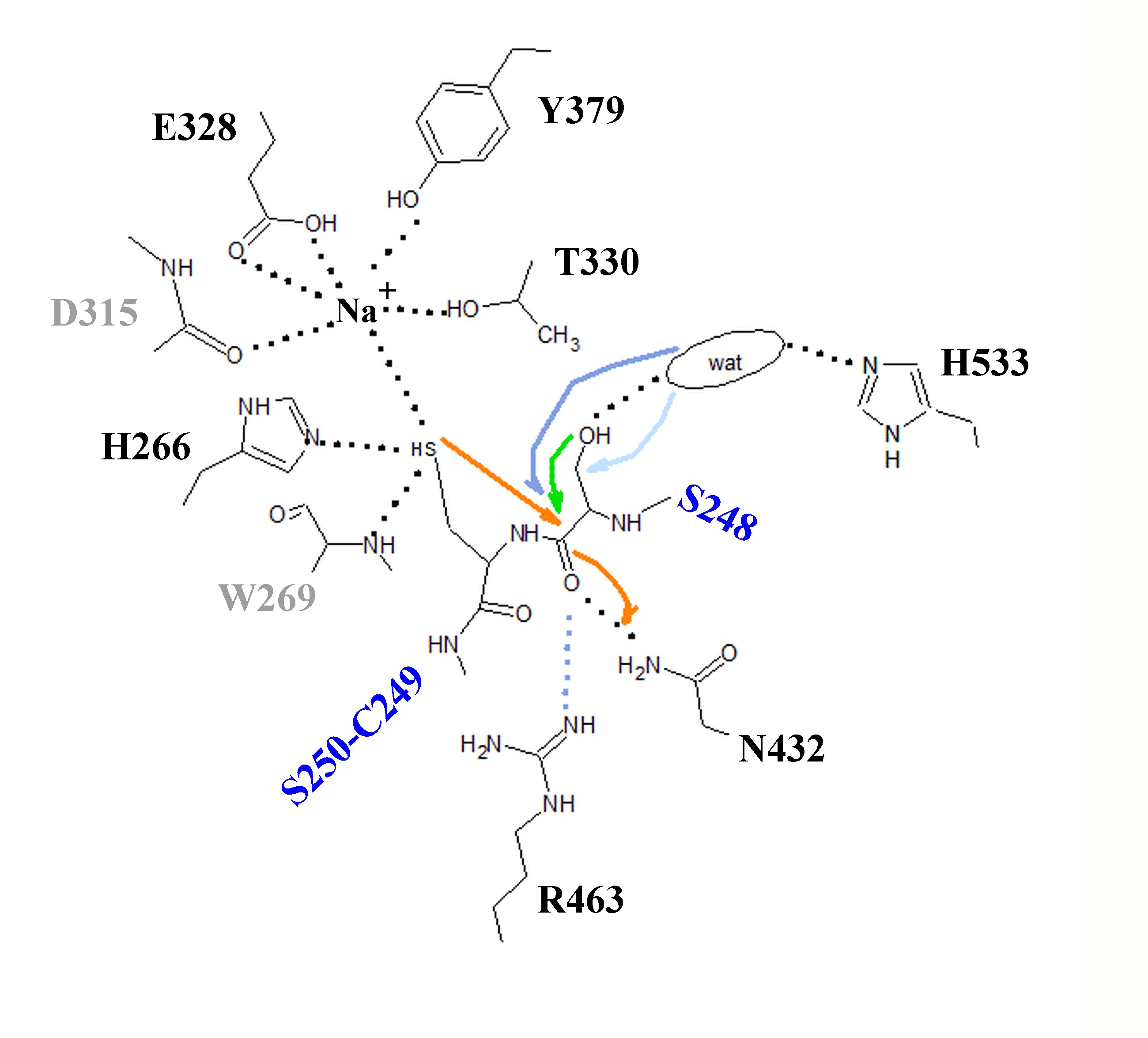

Supplement: Additional file 7 — Figure S6. Putative mechanism of the auto-proteolytic cleavage between S248 and C249 during the maturation process of the 66.3 kDa protein. Residues of and adjacent to the scissile peptide bond are labeled in blue, while residues of which side chain and backbone atoms are involved in the represented interactions, are labeled in black and grey, respectively. The first nucleophilic attack at the carbonyl carbon of S248 by the sulfhydryl group of C249 and the subsequent formation of the oxyanion are indicated by orange arrows. Possible attacks following this transition state are represented by green and blue arrows depending on whether the oxygen atom is part of the serine side chain or of a bound water molecule. [file 1472-6807-9-56-S7.jpeg]
