# Supplementary material for: Initial insight into the function of the lysosomal 66.3 kDa protein from mouse by means of X-ray crystallography
Source: BMC Struct Biol. 2009 Aug 25;9:56. doi: 10.1186/1472-6807-9-56 (PMC2739207; doi:10.1186/1472-6807-9-56)
Supplement: Additional file 4 — Table S1. Extended list of structures with a similar fold as the 66.3 kDa protein revealed using the program DALI. [file 1472-6807-9-56-S4.pdf]

Tab. S1. Extended list of structures with a similar fold as the 66.3 kDa protein revealed using the program DALI.

protein	PDB-ID*	Z-score	Rmsd [Å]	L_{ali}	N_{res}	% ID
**Cephalosporin acylase* (CA)**	1oqz	17.0	3.6	360	684	11
**Penicillin V acylase (PVA)**	2pva	16.2	3.0	222	334	6
**Conjugated bile acid (=choloylglycine) hydrolase (CABH)**	2bjf	16.2	3.1	224	328	6
**Penicillin G acylase (PGA)**	1k5s	15.4	3.4	244	557	11
**IMP cyclohydrolase (IMPC)**	2ntm	8.4	3.2	165	202	14
**20 S proteasome**	1ryp	8.3	3.1	161	205	7
**conserved protein#**	1kuu	8.2	3.2	161	202	14
**Proteasome component Y7**	1g0u	8.2	3.0	157	196	8
**Proteasome α subunit**	1j2q	7.8	3.2	154	202	11
**Proteasome α-type subunit 1**	2h6j	7.3	4.1	173	242	16
**HSLV protease**	1g3k	7.2	3.0	141	173	9
**Proteasome component C7-α**	1z7q	7.2	5.6	167	243	11
**ATP-dependent HSL protease ATP-binding subunit**	1ofh	7.2	3.1	141	173	9
**ATP-dependent HSLU protease ATP-binding subunit**	1g3i	7.0	3.4	146	173	9
**Protein YPL144W**	2z5c	6.4	3.3	139	189	13
**UNP Q5LQD5_SILPO (hypothetical protein)**	2imh	6.1	4.1	157	226	17
**Glutamine PRPP amidotransferase**	1gph	2.8	4.3	129	465	10
**Horse plasma gelsolin**	1d0n	2.8	4.1	109	729	7
**Antithrombin III**	1att	2.6	7.8	86	420	5

* cephalosporin acylase = glutarylamidase = glutaryl acylase = glutaryl-7-aminocephalosporanic acid acylase, PRPP = phosphoribosylpyrophosphate, # conserved protein of unknown function (structural genomics)
